# Supplementary material for: Comparative multiomics analysis of cell physiological state after culture in a basket bioreactor
Source: Sci Rep. 2022 Nov 23;12:20161. doi: 10.1038/s41598-022-24687-4 (PMC9686226; doi:10.1038/s41598-022-24687-4)
Supplement: Supplementary file 1 — Supplementary Information 1. [file 41598_2022_24687_MOESM1_ESM.zip › raw data/Proteomic raw data/6.KEGGenrichment/EG.vs.CG/ALL/pathway/src/04514.html]

map04514


- K03160

- Down regulated proteins

A0A0D9RQI3(Description:B-cell surface antigen CD40;Gene:CD40;log2FC:-1.3133)

- K03160

- Down regulated proteins

A0A0D9RQI3(Description:B-cell surface antigen CD40;Gene:CD40;log2FC:-1.3133)

- K03160

- Down regulated proteins

A0A0D9RQI3(Description:B-cell surface antigen CD40;Gene:CD40;log2FC:-1.3133)

- K03160

- Down regulated proteins

A0A0D9RQI3(Description:B-cell surface antigen CD40;Gene:CD40;log2FC:-1.3133)

- K03160

- Down regulated proteins

A0A0D9RQI3(Description:B-cell surface antigen CD40;Gene:CD40;log2FC:-1.3133)

- K05693

- Down regulated proteins

A0A0D9RYA9(Description:Protein-tyrosine-phosphatase;Gene:PTPRM;log2FC:-1.3184)

- K05693

- Down regulated proteins

A0A0D9RYA9(Description:Protein-tyrosine-phosphatase;Gene:PTPRM;log2FC:-1.3184)

- K06759

- Down regulated proteins

A0A0D9R457(Description:Uncharacterized protein;Gene:CNTN1;log2FC:-inf)

- K06759

- Down regulated proteins

A0A0D9R457(Description:Uncharacterized protein;Gene:CNTN1;log2FC:-inf)

- K06759

- Down regulated proteins

A0A0D9R457(Description:Uncharacterized protein;Gene:CNTN1;log2FC:-inf)

- K06759

- Down regulated proteins

A0A0D9R457(Description:Uncharacterized protein;Gene:CNTN1;log2FC:-inf)

- K06759

- Down regulated proteins

A0A0D9R457(Description:Uncharacterized protein;Gene:CNTN1;log2FC:-inf)

- K06781

- Down regulated proteins

A0A0D9S368(Description:Uncharacterized protein;Gene:CADM1;log2FC:-1.5510)

- K06781

- Down regulated proteins

A0A0D9S368(Description:Uncharacterized protein;Gene:CADM1;log2FC:-1.5510)

- K06781

- Down regulated proteins

A0A0D9S368(Description:Uncharacterized protein;Gene:CADM1;log2FC:-1.5510)

- K06781

- Down regulated proteins

A0A0D9S368(Description:Uncharacterized protein;Gene:CADM1;log2FC:-1.5510)

- K06491

- Down regulated proteins

A0A0D9S2Z2(Description:Neural cell adhesion molecule 1;Gene:NCAM1;log2FC:-1.7712)

- K06491

- Down regulated proteins

A0A0D9S2Z2(Description:Neural cell adhesion molecule 1;Gene:NCAM1;log2FC:-1.7712)

- K06491

- Down regulated proteins

A0A0D9S2Z2(Description:Neural cell adhesion molecule 1;Gene:NCAM1;log2FC:-1.7712)

- K06491

- Down regulated proteins

A0A0D9S2Z2(Description:Neural cell adhesion molecule 1;Gene:NCAM1;log2FC:-1.7712)

- K06550

- Down regulated proteins

A0A0D9R3G8(Description:Uncharacterized protein;Gene:L1CAM;log2FC:-1.8114)

- K06550

- Down regulated proteins

A0A0D9R3G8(Description:Uncharacterized protein;Gene:L1CAM;log2FC:-1.8114)

- K06550

- Down regulated proteins

A0A0D9R3G8(Description:Uncharacterized protein;Gene:L1CAM;log2FC:-1.8114)

- K06550

- Down regulated proteins

A0A0D9R3G8(Description:Uncharacterized protein;Gene:L1CAM;log2FC:-1.8114)

- K06527

- Down regulated proteins

A0A0D9S6M6(Description:Uncharacterized protein;Gene:VCAM1;log2FC:-1.9990)

- K06735

- Up regulated proteins

A0A0D9R9F6(Description:Uncharacterized protein;Gene:JAM2;log2FC:1.1538)

- K06735

- Up regulated proteins

A0A0D9R9F6(Description:Uncharacterized protein;Gene:JAM2;log2FC:1.1538)

- K06735

- Up regulated proteins

A0A0D9R9F6(Description:Uncharacterized protein;Gene:JAM2;log2FC:1.1538)

- K06746

- Down regulated proteins

A0A0D9RNI2(Description:Ig-like domain-containing protein;Gene:;log2FC:-1.1514)

- K06710

- Down regulated proteins

A0A0D9R6A4(Description:Uncharacterized protein;Gene:;log2FC:-inf)

Close
